# Understanding and controlling asthma in Latin America: A review of recent research informed by the SCAALA programme

**DOI:** 10.1002/clt2.12232

**Published:** 2023-03-26

**Authors:** Philip J. Cooper, Camila A. Figueiredo, Alejandro Rodriguez, Leticia Marques dos Santos, Rita C. Ribeiro‐Silva, Valdirene Leao Carneiro, Gustavo Costa, Thiago Magalhães, Talita dos Santos de Jesus, Raimon Rios, Hugo Bernardino F. da Silva, Ryan Costa, Martha E. Chico, Maritza Vaca, Neuza Alcantara‐Neves, Laura C Rodrigues, Alvaro A. Cruz, Mauricio L. Barreto

**Affiliations:** ^1^ Escuela de Medicina Universidad Internacional del Ecuador Quito Ecuador; ^2^ Institute of Infection and Immunity St George's University of London London UK; ^3^ Instituto de Ciências da Saúde Universidade Federal da Bahia Salvador Brazil; ^4^ Departamento de Saúde Coletiva Universidade Federal da Bahia Salvador Brazil; ^5^ Escola de Nutrição Universidade Federal da Bahia Salvador Brazil; ^6^ Departamento de Ciências da Vida Universidade do Estado da Bahia Salvador Brazil; ^7^ Center for Data Knowledge and Integration for Health (CIDACS) Fundação Oswaldo Cruz Bahia Salvador Brazil; ^8^ Universidade Salvador (UNIFACS) Salvador Bahia Brazil; ^9^ Instituto de Saúde Coletiva Universidade Federal da Bahia Salvador Brazil; ^10^ Fundacion Ecuatoriana para la Investigacion en Salud (FEPIS) Esmeraldas Ecuador; ^11^ Faculty of Epidemiology and Population Health London School of Hygiene and Tropical Medicine London UK; ^12^ Universidade Federal da Bahia and Fundação ProAR Salvador Brazil

**Keywords:** allergy, asthma, determinants, Latin America

## Abstract

Asthma is an important health concern in Latin America (LA) where it is associated with variable prevalence and disease burden between countries. High prevalence and morbidity have been observed in some regions, particularly marginalized urban populations. Research over the past 10 years from LA has shown that childhood disease is primarily non‐atopic. The attenuation of atopy may be explained by enhanced immune regulation induced by intense exposures to environmental factors such as childhood infections and poor environmental conditions of the urban poor. Non‐atopic symptoms are associated with environmental and lifestyle factors including poor living conditions, respiratory infections, psychosocial stress, obesity, and a diet of highly processed foods. Ancestry (particularly African) and genetic factors increase asthma risk, and some of these factors may be specific to LA settings. Asthma in LA tends to be poorly controlled and depends on access to health care and medications. There is a need to improve management and access to medication through primary health care. Future research should consider the heterogeneity of asthma to identify relevant endotypes and underlying causes. The outcome of such research will need to focus on implementable strategies relevant to populations living in resource‐poor settings where the disease burden is greatest.

## 1 INTRODUCTION

**FIGURE 1:**
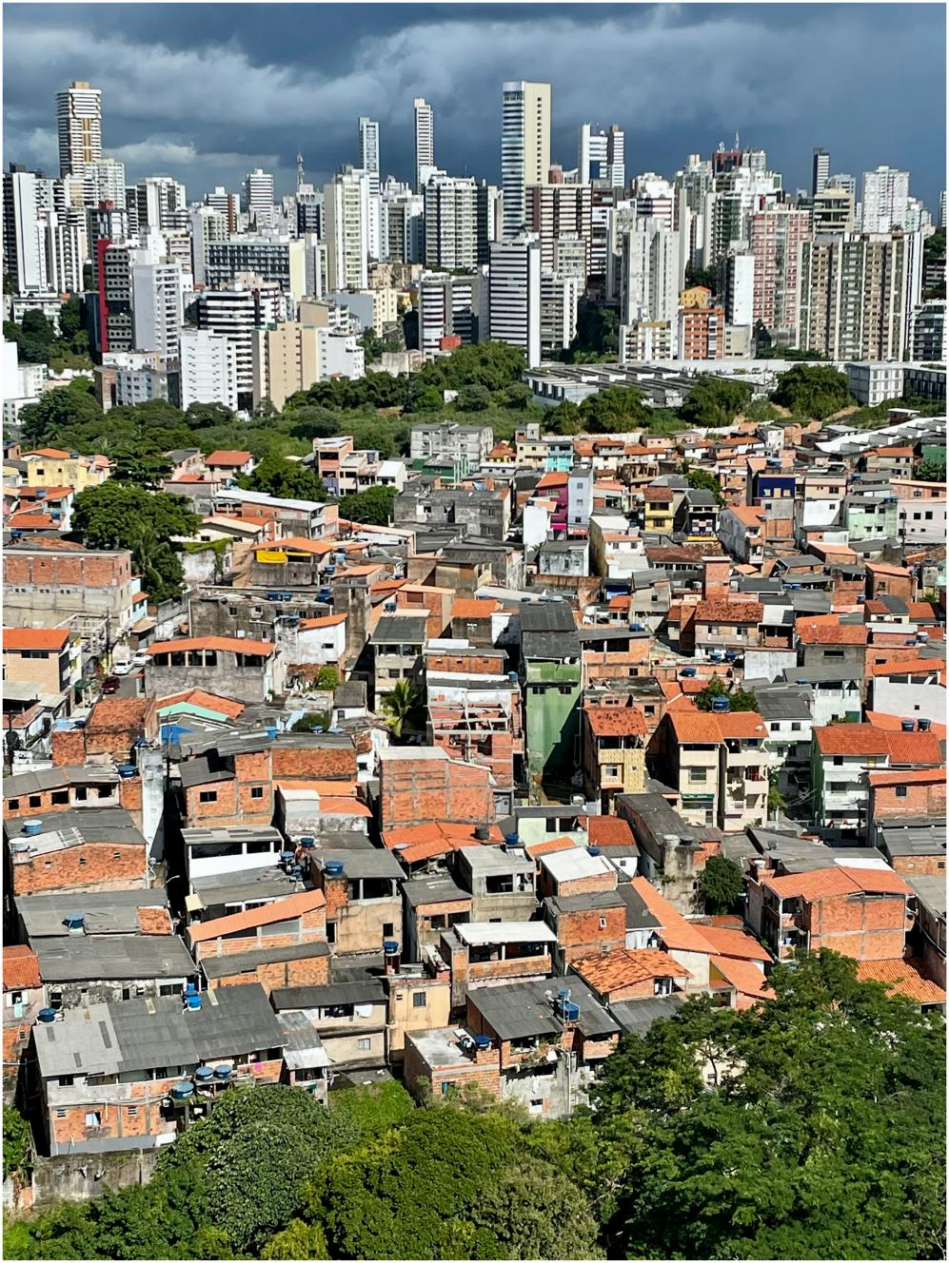
Latin American societies are highly unequal: underserved neighbourhood and affluent high‐rise apartment buildings in the City of Salvador, Brazil.

Asthma is the most common chronic respiratory disease (CRD) worldwide and is estimated to affect 262 million causing significant mortality and morbidity,^1^ and has emerged as an important public health problem in many Latin American (LA) countries over the last 30 or so years. LA is a highly diverse region in terms of geography, climate, wealth, and ethnicity including 20 different countries with 639 million inhabitants, where 40 million are estimated to have asthma.^2^ A common feature of LA countries is the high level of social inequalities^3^ (Figure 1). In LA, asthma prevalence in both children and adults is highly variable and, where high, is among the highest worldwide, particularly in coastal tropical cities.^4^

In the present review, we discuss progress in our understanding of how social, environmental and host genetic factors affect asthma prevalence, morbidity, and mortality in LA with a particular focus on the findings of SCAALA (Social Changes, Asthma, and Allergies in Latin America), an ongoing asthma research programme in Brazil and Ecuador.^5^, ^6^ This review does not consider the specific role of several important exposures such as air pollutants on asthma, or findings in Hispanic populations living in the United States and associated territories, that have been covered comprehensively elsewhere.^7^, ^8^, ^9^, ^10^

## 2 EPIDEMIOLOGY OF ASTHMA AND DISEASE BURDEN

### 2.1 Asthma prevalence

The ISAAC studies (International Study of Asthma and Allergies in Children) reported a high prevalence of asthma symptoms (≥20%) and severe asthma (≥7.5%) in adolescents living in LA cities.^11^ The prevalence of asthma symptoms between countries ranged from 6.6% in Cuernavaca, Mexico, to 27.1% in Salvador, Brazil. Over half of LA countries reported a prevalence of 15% or more in children.^4^ Extensive national surveys of asthma prevalence in representative samples of Brazilian adolescents confirmed a high prevalence of recent symptoms (23.2%) in 2012, ranging from 16.8% in Salvador to 27.3% in Porto Alegre,^12^ but which had declined in a repeat survey in 2015.^13^ Ecological studies of children^14^ and adolescents^15^ across LA cities suggested that social inequalities were a major determinant of asthma prevalence.

### 2.2 Non‐atopic asthma is predominant

A consistent observation across population‐based studies of children with asthma in LA and other low and middle‐income country (LMIC) settings has been the relatively weak associations with atopy. ISAAC phase II estimated approximately 40% of asthma to be attributable to atopy (measured by allergen skin prick test reactivity [SPT] or presence of allergen‐specific IgE [sIgE]) among study centres from affluent countries but only 20% in non‐affluent countries, being approximately 11% in the two LA study centres in rural Ecuador and urban Brazil.^16^ Similarly, low fractions of childhood asthma attributable to sIgE (population attributable fraction [PAF%], 24.5%) were observed in Salvador, Brazil,^17^ and to SPT and sIgE in urban (10.7% vs. 26.0%) and rural (3.9% vs. 10.5%) coastal Ecuador.^18^, ^19^ Atopy was more strongly associated with asthma (PAF% of 68.5% for sIgE and 57.2% for SPT) among children presenting with asthma attacks to Emergency Rooms in urban Ecuador,^20^ indicating a link with disease severity.^21^ A predominance of non‐atopic asthma in population samples of children has been observed in other LMIC settings.^16^

### 2.3 Disease burden

Asthma was estimated to cause 461,000 deaths worldwide in 2019 with an overall burden of disease of 21.6 million DALYs.^1^ Age‐standardized DALY rates in LA have remained stable since 1990.^22^ However, mortality and hospitalization rates from asthma have declined in Brazil since the 1990s,^23^ and in Ecuador^24^ and Costa Rica^25^ since 2000. Such time trends likely reflect improved health access and availability of asthma medications.^26^

## 3 ENVIRONMENT

### 3.1 Infections, poor hygiene, and allergic inflammatory responses

#### 3.1.1 The hygiene hypothesis and LA

The hygiene hypothesis was proposed to explain temporal trends of increasing prevalence of asthma and allergic diseases.^27^ Such trends were explained by reduced exposures to infectious diseases and environmental microbes during early childhood. The hypothesis was believed to operate through immunological mechanisms in which early life microbial exposures provided key signals for the maturation of the immune response—children living in more hygienic circumstances were considered to receive insufficient microbial signals resulting in delayed immune maturation and the induction of less robust immune regulation making them more vulnerable to inflammatory diseases such as asthma.^28^, ^29^ Similarly, children born and brought up on traditional European farms had a reduced risk of atopy and asthma compared to children not living on farms, and this protection was attributed to intense exposures to a wide diversity of microbial products such as endotoxin on farms.^30^, ^31^, ^32^ The observations of a greater risk of asthma in poor inner‐city neighbourhoods in the United States and in marginalized urban populations in LA where, presumably, hygienic conditions were less than optimal, was taken as evidence against a role for this hypothesis.^33^

#### 3.1.2 Poor hygiene exposures and immune regulation

Initial observations from underprivileged neighbourhoods in Salvador, Northeast Brazil, showed that poor environmental hygiene (such as living in a household without a sewage or tap water connection, and unpaved streets)^34^ and being infected with geohelminth parasites were associated with greater in vitro production of the immune regulatory cytokine, IL‐10, by peripheral blood leucocytes (PBLs).^35^, ^36^, ^37^ Elevated IL‐10 regulated both Th1 and Th2 responses in this population. Studies in Ecuador of children living in traditional riverine communities and underserved urban neighbourhoods in the coastal Province of Esmeraldas showed that IL‐10 production by PBLs was greater in urban children ^36^, ^37^ and that poor hygiene exposures, including geohelminth infections, were associated with cytokine response phenotypes suggestive of a hyporesponsive innate and modified Th2 (an immune response defined by the presence of both Th2 cytokines and IL‐10) adaptive responses. The hyporesponsive innate phenotype was associated with reduced SPT.^37^ Interestingly, there was evidence of a dose‐response effect of helminth infection such that an increasing number of helminth species infecting children was associated with increased IL‐10 but reduced SPT.^38^ Together these data indicate that poor hygiene exposures, likely to be most intense in poor urban neighbourhoods, are associated with increased immune regulation that minimizes allergic inflammatory responses (e.g SPT) but with minimal effects on asthma symptoms.

#### 3.1.3 Parasites and asthma/atopy

Longitudinal analyses in both urban Salvador and rural coastal Ecuador showed that early life infections with geohelminths, particularly *Trichuris trichiura*, were associated with a reduced prevalence of SPT later in childhood.^39^, ^40^ Effects of geohelminths on asthma symptoms have been less consistent. In general, strong effects on prevalence of asthma symptoms have not been observed in cross‐sectional analyses.^37^, ^41^ However, longitudinal analyses of children in rural Ecuador showed contrasting parasite‐specific effects on risk of asthma symptoms in later childhood, with early childhood *T*. *trichiura* decreasing and *Ascaris lumbricoides* increasing risk.^24^ There is strong evidence that allergic sensitization to *Ascaris* (measured by the presence of anti‐*Ascaris* IgE) is associated with asthma symptoms and disease severity.^19^, ^43^, ^44^, ^45^, ^46^, ^47^ The presence of anti‐*Ascaris* IgE explained a higher frequency of asthma symptoms in rural compared to urban children (PAF%, 49.7 vs. 39.4%).^19^ In the case of urban children, particularly those with more severe illness, a much higher proportion of symptoms was explained by mite rather than *Ascaris* sensitization.^17^, ^45^ The observations that *A*. *lumbricoides* can cause severe lung disease in murine models^48^, ^49^ and that IgG seropositivity to *Ascaris* spp. in males living in Northern Europe is associated with impaired lung function,^50^ indicate the need for studies addressing potential effects of ascariasis on CRD in endemic settings in LA.

#### 3.1.4 Common childhood infections and asthma/atopy

An analysis of exposures to childhood infectious diseases among children in Salvador showed that seropositivity to a variety of common bacteria, viruses, and parasites including active geohelminth infections, were inversely associated with SPT but not associated with reduced asthma symptoms.^51^ Further, a greater burden of common childhood infections in this population and markers of poor environmental hygiene (e.g. low maternal education and unpaved streets) were associated with an under‐responsive cytokine phenotype characterized by low or negligible production of Th1, Th2, and Treg cytokines and a lower prevalence of SPT.^48^ There was evidence that this effect was mediated by cytokine phenotype, but immune response phenotypes and poor hygiene exposures were not associated with asthma symptoms.^52^

These findings show clearly that poor hygiene exposures, including childhood infections, induce immune phenotypes in children that reduce inflammatory responses and the risk of allergic sensitization as indicated by a positive SPT but have little impact on asthma symptoms. The predominance of non‐atopic asthma in LA populations, likely mediated by distinct causal mechanisms to that of atopic disease, could explain a high prevalence of symptoms despite intense unhygienic exposures. In fact, such exposures have been associated with an increased risk of non‐atopic wheeze in these populations.^17^, ^41^, ^42^

### 3.2 Urbanization and migration

#### 3.2.1 Urbanization and asthma

Urbanization refers to the complex and gradual processes by which populations become urban and includes population growth by migration and natural increases resulting in greater population densities, improvements in built infrastructure, and changes in social and economic activities and lifestyles.^53^ The LA population is now predominantly urban (overall 81%), a proportion that is much greater in Brazil [87%] than Ecuador [64%].^54^ The study of the effects of urbanisation on asthma is complex because of the multidimensional nature of this process and the methodological and conceptual limitations in defining and measuring urbanisation.

Much of the evidence supporting a relationship between urbanisation and asthma is derived from prevalence studies comparing rural and urban populations.^39^ Such studies cannot disentangle specific urbanization features linked to asthma.^55^ Despite such limitations, a recent meta‐analysis of observational studies comparing asthma prevalence between urban and rural populations within LMICs estimated an approximately 50% greater prevalence (pooled Odds ratio 1.46) in urban populations irrespective of how asthma was defined.^39^

**FIGURE 2:**
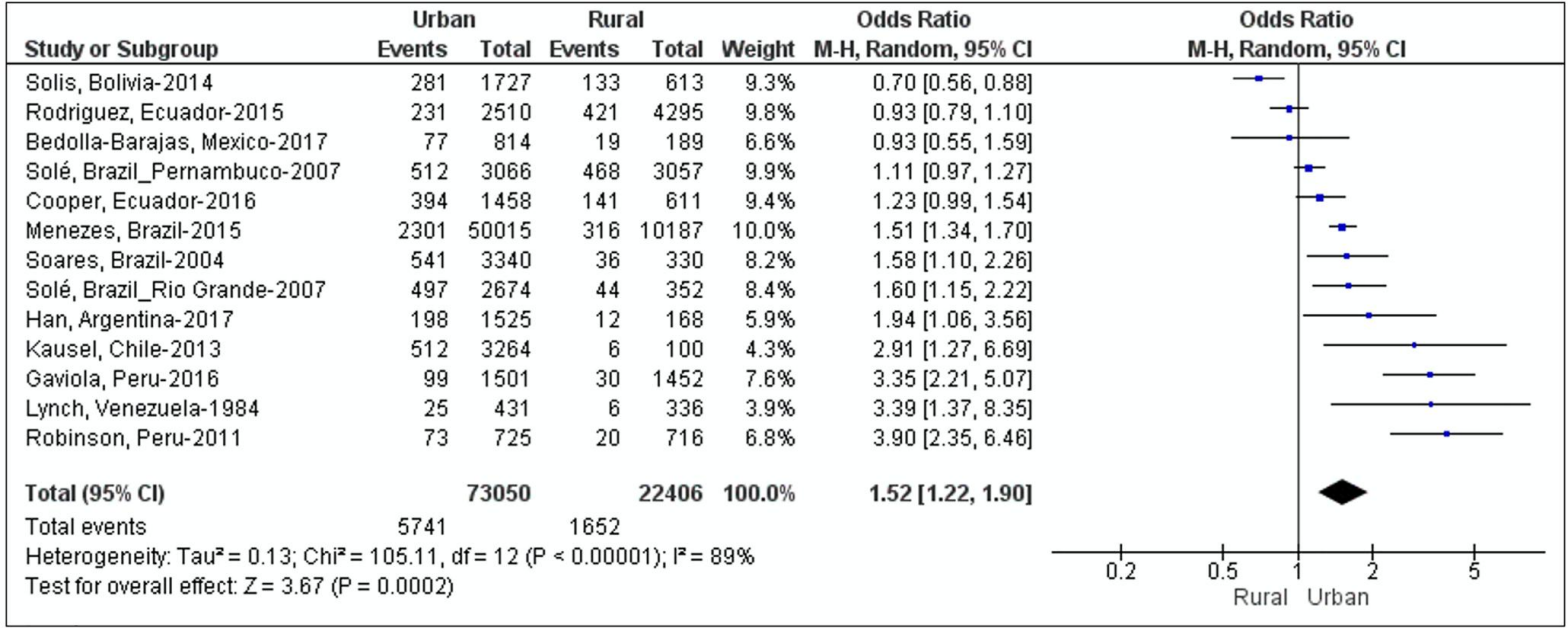
Forest plot of 13 observational studies comparing asthma prevalence between urban and rural populations in Latin America. Shown is a pooled Odds ratio across studies of 1.52.

Overall, 21 studies have evaluated the relationship between urbanisation and asthma in LA. Of these, 13 compared asthma prevalence between urban and rural populations^25^, ^39^, ^56^, ^57^, ^58^, ^59^, ^60^, ^61^, ^62^, ^63^, ^64^, ^65^ (Figure 2), 6 compared asthma prevalence between cities,^14^, ^15^, ^17^, ^39^, ^66^, ^67^ and 2 evaluated intra‐urban variations in asthma.^68^, ^69^ All these studies indicate that urbanisation and internal migration are important determinants of asthma prevalence.

#### 3.2.2 Multidimensional measures of urbanization and urbanicity

In urban studies, an alternative is to use a multidimensional approach to define urbanisation using composite measure of urbanicity (i.e., presence of conditions that are more common in urban areas at any given time)^53^ based on indicators such as urban infrastructure, socioeconomic characteristics, and demographic and geographic features. Two asthma‐related studies (an ecological and a cross‐sectional study) have used this approach in transitional populations in Ecuador,^39^, ^70^ and showed an increasing asthma prevalence with greater levels of urbanisation.

**FIGURE 3:**
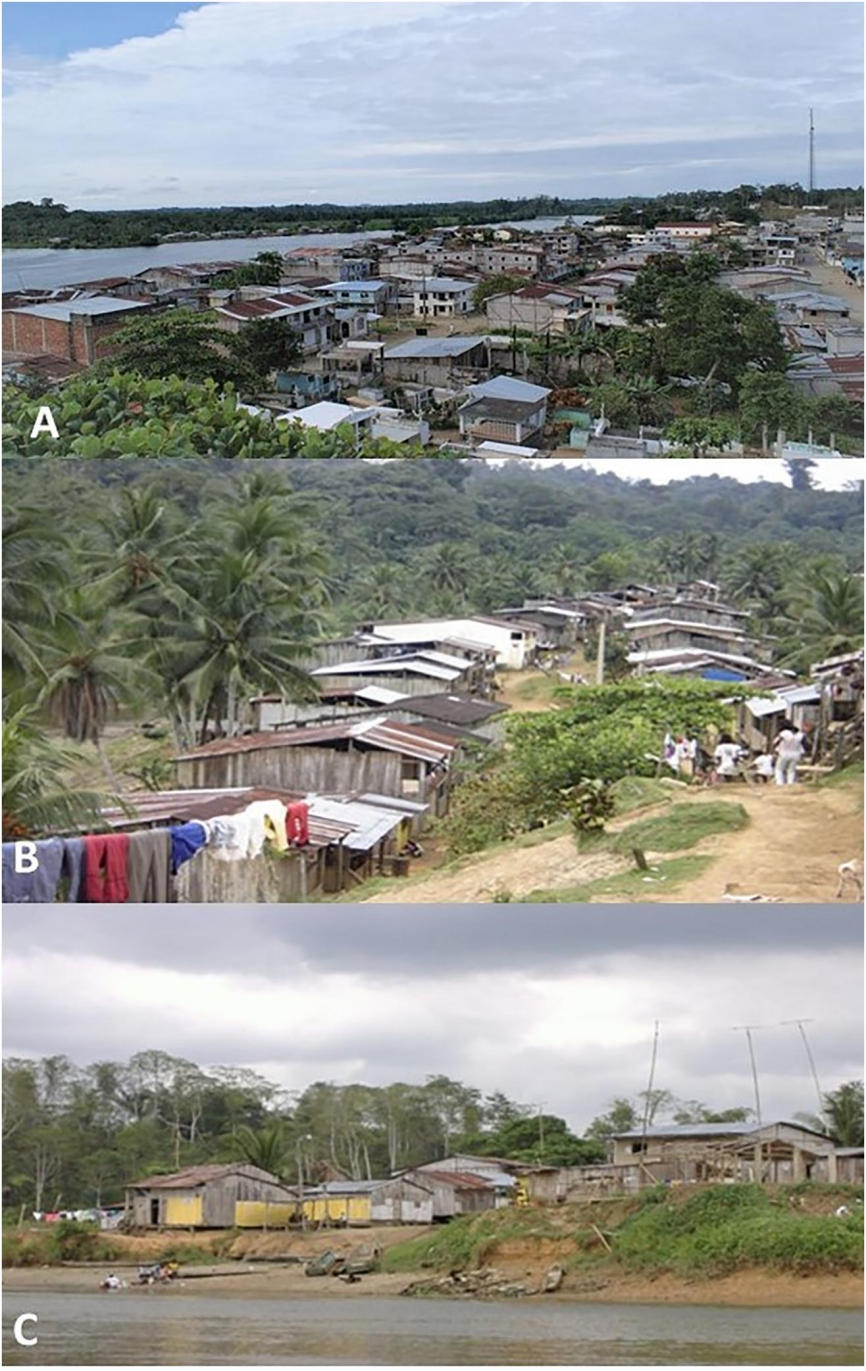
Urbanization processes extend into rural spaces. Examples from Esmeraldas province, Ecuador, are shown. A—more highly urbanised (rural) communities are characterised by a higher population density with neighbourhoods and provision of urban services such as electricity, piped water, roads, and socioeconomic and lifestyle indicators not dependent on agricultural activities. B ‐ medium urbanised communities are characterised by lower population density, limited services but generally electricity, few houses constructed with cement, and an economy and lifestyle largely dependent on agricultural activities. C ‐ Low urbanised communities are characterised by low population density, use of traditional materials for housing, no urban services or road access, and an exclusively agricultural economy.

The process of urbanisation has a profound impact on the lifestyle of populations not only in urban but also in rural areas (Figure 3). Changes in, for example, dietary patterns, health behaviours, work activities, economic status, and housing materials, are all related to the urbanisation process.^71^ Although lifestyle changes have been associated with increased asthma and differences in asthma prevalence between rural and urban populations, it has been difficult to disentangle independent effects of individual risk factors that together constitute lifestyle. To overcome this limitation, an analysis of childhood asthma in Ecuador in which lifestyle was defined as a set of attributes representing groups of linked risk factors, identified lifestyle domains relating to home infrastructure and sedentarism as being most strongly associated with wheeze in urban and rural areas.^72^

#### 3.2.3 Internal migration

Internal migration is a key process contributing to urbanisation that asthma studies in LIMCs have largely overlooked. The migration process, especially from rural to urban areas, involves changes in exposures to environmental and social factors such as pollution, housing, diet, and accessibility to health services, all of which are potential determinants of asthma. Studies in Ecuador have considered the effects of internal migration on asthma prevalence: cross‐sectional analyses have evaluated migration patterns in rural and urban areas and showed that children living in an urban area with a history of rural migration had a higher prevalence of asthma compared with the non‐migrant population,^73^, ^74^ while a higher prevalence of childhood asthma in rural areas was associated with the absence of the mother. Mothers migrate to urban areas searching for work, leaving their children to be cared for by other family members.^73^ These observations illustrate how social patterns associated with rural‐urban migration, such as the feminization of migration and internal migration may contribute to temporal and geographical differences in asthma prevalence between urban and rural areas.

## 4 LIFESTYLE AND BEHAVIOUR

### 4.1 Diet, asthma, and allergies

#### 4.1.1 Obesity

Obesity is increasing fast in LA countries.^75^, ^76^ Although studies in different settings have shown an association between asthma and obesity,^77^, ^78^ the causal nature of this association is unclear: obesity may trigger or aggravate wheezing/asthma, while wheezing/asthma may also contribute to obesity through, for example, reduced physical activity.^79^ However, longitudinal studies indicate that obesity precedes asthma onset.^80^ Similar observations have been reported in Brazil^81^, ^82^ and elsewhere in LA.^83^, ^84^ Obesity‐related asthma has been linked to non‐eosinophilic phenotypes (based on the pattern of inflammatory cells in induced sputum) that tend to be refractory to treatment.^85^ An analysis in Brazilian children indicated that obese asthmatics were more likely to have non‐atopic disease.^81^

#### 4.1.2 Dietary patterns

Adopting a so‐called Western lifestyle includes changes in diet and increased consumption of highly processed foods.^86^, ^87^ Studies of dietary patterns and asthma symptoms in Brazilian populations have shown that a Westernized diet, characterized by high levels of saturated fats and low omega‐3 polyunsaturated fatty acid (n‐3‐PUFA), is associated with increased asthma risk. Suboptimal dietary intake of antioxidant vitamins, particularly vitamins A, C, and E, and carotenoids, and other antioxidants such as selenium, zinc and flavonoids, may adversely affect the modulation of oxidative lung stimuli.^86^, ^88^ In contrast, higher intakes may have beneficial effects on the modulation of oxidative lung stimuli, thus decreasing airway hyperactivity, wheezing symptoms, and asthma.^89^ For example, low serum zinc levels were associated with a doubling of wheeze prevalence.^90^ Inverse associations have been demonstrated between a Mediterranean dietary pattern and asthma in Mexican children,^91^ while fish consumption was associated with reduced risk of asthma symptoms, particularly non‐atopic wheeze/asthma.^82^ A recent study in children in Puerto Rico provided evidence that an effect of healthier diet in reducing asthma risk might be associated with a reduction in plasma levels of the inflammatory cytokine, IL‐17‐F^92^

### 4.2 The role of stress and other psychosocial problems on asthma/allergy

#### 4.2.1 Mental illness and psychological stress

There is a well‐established relationship between psychological factors and asthma.^87^ Studies in LA have reinforced the role of stress and other psychosocial problems as determinants of asthma prevalence (Table 1). In Brazil, asthma symptoms were more frequent in children of mothers with common mental disorders in a setting where children were typically exposed to intrafamilial and community violence and had a high frequency of behavioural problems often accompanied by depression, anxiety, and stress.^93^, ^94^, ^95^, ^96^, ^97^, ^98^, ^99^ Interestingly, maternal mental illness predicted the development of asthma symptoms in children and adolescents.^100^ Asthma itself may also increase the risk of mental disorders.^101^

**TABLE 1:** Summary of findings of associations between psychosocial stress and asthma in Latin America.

Stress condition	Asthma status	OR (CI: 95%)	Population	Locality	Reference
Common mental disorders in the mothers (e.g., anxiety, depression and maternal stress)	Asthma	1.78 (1.34–2.35)	Mothers and children aged 5–12 years, (SCAALA)	Salvador, Brazil	94
	Atopic	1.74 (1.12–2.71)	Children aged 4–12 years (SCAALA)	Salvador, Brazil	95
	Non‐atopic	1.73 (1.17–2.55)			
	Asthma	1.13 (1.02–1.25)	Twins age 1 year (PRINTS)	Puerto Rico	171
		1.13 (1.01–1.27)	Twins age 3 years (PRINTS)		
Behavioural problems (e.g., somatic complaints, anxious/depressed, social problems, thought problems, attention problems, and aggressive behaviour)	Asthma	1.40 (1.08–1.81)	Children aged 6–12 years (SCAALA)	Salvador, Brazil	97
Depression/anxiety	Wheezing remission in asthmatic children	0.47 (0.25–0.94)	Children aged 4–12 years (SCAALA)	Salvador, Brazil	99
Postnatal stress	Current wheeze	1.21 (1.08–1.35)	Children aged 4 years (PROGRESS)	Mexico City, Mexico	172
	Ever wheeze	1.12 (1.04–1.21)			
Perceived discrimination	Asthma	1.65 (1.09–2.50)	Mexican Americans (low socioeconomic status) aged 8–21 years (GALA II)	Living in United States and Puerto Rico	173
		3.09 (1.03–10.1)	Latin children (high socioeconomic status) aged 8–21 years (GALA II)		
Suicide attempt	Asthma	3.01 (1.80–5.01)	Adults aged 18–64 years	Puerto Rico	103
Nonviolent discipline (e.g., explained why something was wrong, ‘time‐out’, gave him something else to do instead of what he was doing, and took away privileges or grounded him)	Non‐atopic	1.95 (1.17–3.25)	Children aged 4–12 years (SCAALA)	Salvador, Brazil	98
Maltreatment nonviolent discipline (e.g., corporal punishment and psychological aggression dimensions)	Non‐atopic	1.95 (1.19–3.20)	Children aged 4–12 years (SCAALA)	Salvador, Brazil	98
Intrafamilial and community violence					
Physical aggression committed by an adult in the family	Asthma	1.38 (1.33–1.43)	Students aged 13–15 years (PeNSE)	Brazil, national	13
Knew someone who had either been beaten or injured with a firearm or knife	Asthma	1.41 (1.03–1.93)	Children aged 4–12 years (SCAALA)	Salvador, Brazil	96
Awareness of gang warfare or drug traffic in the neighbourhood		1.39 (1.01–1.92)			
Heard a gunshot more than once/gun violence exposure	Atopic	1.72 (1.03–2.89)	Children aged 6–14 years	San Juan, Puerto Rico	174
	Asthma	1.80 (1.10–1.70)	Children aged 9–14 years		175
Exposure to violence	Asthma	1.14 (1.07–1.21)	Adults aged 18–64 years	Puerto Rico	103
Exposure to violence/methylation of *ADCYAP1R1*	Asthma (associated with methylation of *ADCYAP1R1*)	0.05^a^ (0.01–0.09)	Children aged 9–14 years	San Juan, Puerto Rico	148
≥3 Childhood abuse events (physical and/or sexual)	Asthma	1.88 (1.06–3.34)	Pregnant women aged 18 years and older (PrOMIS)	Lima, Peru	176

Abbreviations: CI, Confidence interval; GALA II, Genes‐Environment and Admixture in Latino Americans; OR, Odds ratio; PeNSE, National Adolescent School‐Based Health Survey; PRINTS, Puerto Rican Infant Twin Study; PROGRESS, Programing Research in Obesity, Growth, Environment and Social Stressors; PrOMIS, Pregnancy Outcomes, Maternal and Infant Study; SCAALA, Social Change, Asthma and Allergy in Latin America.

^a^ Beta value.

Multiple social and stress factors such as inequalities in health care access, exposure to stressors such as personal or community violence, individual and parental mental illness or behavioural problems^84^, ^96^, ^97^, ^102^, ^103^ could contribute to asthma risk. LA is the most urbanized, unequal, and violent region globally, and where some of the highest prevalence rates for childhood asthma have been reported.^104^ Associations between several mental disorders and asthma have been reported in LA populations.^105^, ^106^ A study of adults from São Paulo, Brazil, showed that anxiety with or without depression was associated with uncontrolled asthma.^107^ A longitudinal analysis of asthmatic children in Salvador, Brazil, showed a reduced chance of remission and greater risk of developing severe symptoms among children internalizing problems.^99^ Policies to prevent domestic and community violence can be justified on public health grounds because of the detrimental effects on mental health and asthma risk.^108^

#### 4.2.2 Food and nutrition insecurity

Food and nutrition insecurity (FNI) has been linked to psychological distress and symptoms of maternal depression, which impact children negatively.^109^, ^110^ Associations of an increased risk of asthma with greater FNI have been reported in northeastern Brazil.^111^ FNI is a chronic source of stress that may contribute to children's emotional and behavioural problems, such as anxiety, depression, and behavioural disorders which themselves can trigger or worsen asthma symptoms.^109^

## 5 HOST GENETICS AND ANCESTRALITY

Populations in LA have high levels of genetic admixture derived from mixing indigenous populations through waves of migration from Europe and forced migrations from Africa since the 16th Century.^112^ Population genetics and ethnic‐racial characteristics differ greatly across countries in the region and likely contribute to differences in risk of asthma and allergy.

### 5.1 Immune genes and asthma/atopy

Studies from Brazil have addressed the role of immune‐related genes in determining risk of asthma and allergy in a population of children living in an underprivileged urban setting in Salvador, Brazil, using a candidate gene approach and showed that: (1) variants in IL‐10 gene were positively associated with risk of atopic wheeze and markers of allergy while being inversely associated with IL‐10 production^113^; (2) variants in the *TGFB1* gene were related to a lower risk of developing allergy^114^; and (3) variants in other immune‐related genes were shown to be associated with asthma and/or atopy.^115^, ^116^, ^117^, ^118^, ^119^, ^120^ Findings are summarized in Table 2.

**TABLE 2:** Summary of findings of associations between genetics and asthma in Latin America.

SNP	Gene or Region	Study	Population	Association with asthma outcome	Reference
rs4410198	*LOC*107985322	*GWAS*	Peru and Brazil (SCAALA)	Positive, lung function	127
rs822396	*ADIPOQ*	*Candidate gene*	Brazil (SCAALA)	Positive	147
rs1063537				Positive	
rs11763517	*LEP*			Negative	
rs11760956				Negative	
rs3024491	*IL*10	*Candidate gene*	Brazil	Association, severity	177
rs9279	*VDR*	*Candidate gene*	Brazil (SCAALA)	Negative	178
rs4328262				Association, severity	
rs2189480				Association, severity	
rs4065275	17*q*12‐21 *locus*	*Candidate gene*	Brazil (SCAALA)	Association, childhood asthma	118
rs12603332					
rs73985228					
rs77777702					
rs1974226	*IL*17*A*	*Candidate gene*	Brazil (SCAALA)	Positive	179
rs279548					
rs1323556	*CYSLTR*2	*Candidate gene*	Brazil (SCAALA)	Negative	117
rs1575464				Negative	
rs61735175				Association, severity	
rs13277810	LOC101927815	*GWAS*	Latin America and Caribbean (CAAPA)	Positive	129
rs114647118	TATDN1			Negative	
rs3122929	STAT6, LRP1 (intergenic)			Positive	
rs10519067	RORA			Negative	
rs907092	17q12–21 *locus*			Negative	
deletion at 6p22.1	6p22.1	*Candidate gene*	Brazil (SCAALA)	Association	118
rs1681577	*DAD1*	*Candidate gene*	Brazil (SCAALA)	Negative	116
rs4981436	*OXA1L*			Positive	
rs2601796	*ADCY*9	*Candidate gene*	Brazil (SCAALA)	Positive	115
rs2601814				Positive	
rs12007907	*IL1RAPL*	*Candidate gene*	Brazil (SCAALA)	Negative	180
rs1800470	*TGFB*1	*Candidate gene*	Brazil (SCAALA)	Negative, allergic asthma	114
rs12551256	*IL33*	*Candidate gene*	Brazil (SCAALA)	Negative	181
rs6691216	*DENND*1*B*	*Candidate gene*	Brazil (SCAALA)	Negative	182
rs1421396				Positive	
rs1421389				Positive	
rs1999071	14q11 locus	*GWAS*	Brazil (SCAALA)	Positive	125
rs1999071	15q22 locus				
rs8029377					
rs3024492	*IL*10	*Candidate gene*	Brazil (SCAALA)	Positive, allergic asthma	113

Abbreviations: ADCY9, Adenylate Cyclase 9 gene; ADIPOQ, Adiponectin gene; CAAPA, Consortium on Asthma among African ancestry Populations in the Americas; CYSLTR2, Cysteinyl Leukotriene Receptor 2 gene; DAD1, (Defender Against Cell Death 1 gene; DENND1B, DENN Domain Containing 1B gene; GWAS, Genome Wide Association Study; IL10, Interleukin 10 gene; IL10, Interleukin 10 gene; IL17A, Interleukin 17 A gene; IL1RAPL, Interleukin 1 Receptor Accessory Protein Like 1; IL33, Interleukin 33 gene; LEP, Leptin gene; LRP1, Low density lipoprotein receptor‐related protein; OXA1L, Mitochondrial Inner Membrane Protein; PROAr, Programme for Control of Asthma in Bahia; RORA, Nuclear receptor ROR alpha gene; SCAALA, Social Change, Asthma, and Allergy in Latin America; STAT6, Signal Transducer And Activator Of Transcription 6; TATDN1, TatD DNase Domain Containing 1 gene; TGFB1, transforming growth factor‐beta 1 gene; VDR, Vitamin D Receptor gene.

### 5.2 GWAS and asthma/atopy

Genome Wide Association Study (GWAS) analyses have primarily focussed on populations of European descent, and it is important that genetically admixed populations such as those found in LA are better represented in the literature. Of all GWAS analyses, only 0.31% have been done in Hispanic or LA populations, while greater than 90% have been done in European‐derived populations.^121^, ^122^ Of 180 studies with asthma as the main outcome, only 15 included Latin Americans.^123^ Greater diversity among participants of genetic studies will improve our understanding of genetic structure across populations and ensure that genomic studies are more generally representative.^118^, ^121^, ^124^, ^125^

An important unresolved question was whether the genetic variants linked to asthma in HICs were also important in high prevalence populations in LA. The first GWAS done in an LA population identified two novel regions of the genome to be associated with asthma symptoms, 14q11 and 15q22.^125^ These regions include genes involved in the modulation of inflammatory and immunological responses, such as LTB4 (leukotriene beta 4 receptor) TRA (T cell alpha receptor) and FOXB1 (forkhead box). The GWAS was able to identify significant associations of asthma with intergenic variants between genes *DAD1* and *OXA1L*. A candidate gene analysis of variants in these 2 genes showed associations with asthma, atopy, and Th1 and Th2 cytokines production. Further polymorphisms in both genes affected gene expression levels and *DAD1* was over‐expressed in asthmatics.^116^ Using the same GWAS, variants in the 17q12‐21 locus were analysed, and among these, three on *OMRDL3* and one on *TOP2A* gene were associated with childhood asthma.^126^

### 5.3 GWAS and asthma‐related traits

Genetic associations with lung function using a GWAS approach in LA populations showed associations of lung function with a novel locus on chromosome 19^127^, ^128^ in Peruvian children and with four loci in an analysis of greater than 7000 asthmatics including Brazilians in the Consortium on Asthma among African ancestry Populations in the Americas (CAAPA). The latter analysis also identified two novel loci (8p23 and 8q24) that may be specific for asthma in populations with African ancestry.^129^, ^130^ Airways response to short‐acting β2‐adrenergic receptor agonists (SABA) has been shown to vary by ethnicity,^131^ and among Latinos at least 15 genetic variants, including several in the 10q21 locus, have been associated with response to SABA.^132^, ^133^, ^134^ Further, GWAS analyses that included Hispanics/Latinos showed variants associated with asthma exacerbations.^130^, ^131^, ^132^, ^133^, ^135^

### 5.4 Role of African ancestry

The question has been raised of whether genetic determinants of asthma in LA populations are related to a high contribution of African ancestry in population genomes. Although asthma prevalence is low (but increasing) in many African countries, individuals of African ancestry living in HICs suffer high prevalence and morbidity.^136^, ^137^ It has been suggested that individuals of African ancestry might have a higher frequency of immunity‐related genetic variants promoting inflammatory responses through evolutionary adaptation of African ancestors to an environment with a high infectious diseases burden, particularly with parasites.^138^ African ancestry is a risk factor for asthma in Brazilians^139^ and other Latino populations.^136^, ^140^ In a population of children living in Salvador, Brazil, with a high level of African admixture, African ancestry was associated with reduced SPT but increased non‐atopic asthma.^125^ An admixture mapping meta‐analysis that included 5 studies of childhood asthma with almost 4000 LA subjects, showed that ancestry at the locus 18q21 was associated with asthma. Native American ancestry was associated with an increased risk, while European ancestry was associated with protection from asthma.^141^ This analysis also indicated that multiple ancestry‐informative noncoding variants upstream of SMAD2 might have a role in asthma susceptibility. Admixture mapping of Brazilian children identified that African ancestry at the loci 17q21.31, 10q22.2, and 2p23.1 were associated with lower lung function.^126^ Further, equations for predicting lung function in children, based on genetic ancestry have proven to be more accurate than standard models that use ethnic‐racial classification.^142^ The observation in Brazil that African ancestry was negatively associated with maternal education and household income and positively associated with infections^127^ indicates the need for caution in interpreting significant associations of African ancestry with a disease as being indicative of genetic rather than social and environmental factors. Indeed, the role of environmental factors as mediators of the effect of African ancestry on asthma‐related outcomes needs to be considered. For example, family hardship and disease management mediated more than 50% of the effect of African ancestry on paediatric asthma readmission.^143^

### 5.5 Gene‐environment interactions

There is a growing recognition of the importance of gene‐environment interactions as determinants of allergic disease.^144^, ^145^ A genome‐wide interaction study from Puerto Rico showed that dust mite allergen exposure appeared to modify the effect of a variant on chromosome 8q24.13 on FEV_1_ in asthmatic children.^146^ In Brazil, protection against asthma and atopy attributed to variants on the genes for leptin (LEP) and adiponectin (ADIPOQ) were lost in overweight individuals.^147^ A study among Puerto Rican children exposed to violence showed an association between greater methylation of the anxiety regulating gene, *ADCYAP1R1*, and asthma.^148^ A significant interaction in Brazilian children was observed between variants on ORMDL3 and seropositivity for the Varicella zoster virus (i.e., the association with asthma was observed only among seropositives).^149^

## 6 ASTHMA MANAGEMENT AND CONTROL

### 6.1 No real priority despite high burden of disease

LMICs suffer a high proportion of the global morbidity and mortality caused by CRDs,^150^ and CRDs including asthma have been listed for many years as priorities for control by WHO^151^, ^152^ and the United Nations. However, in most LA countries, asthma has never been prioritized, and consequently, has been considered a neglected non‐communicable disease.^153^

### 6.2 Poor asthma control, poor adherence, and severe asthma

Findings of population‐based surveys of asthma control in LA have shown that most asthmatics do not have well‐controlled symptoms. A survey of 2169 asthmatics aged 12 or more years from five LA countries showed that 20% experienced daily symptoms,^154^ and although 60% reported their disease as well‐controlled, only 8% met this criterion while 44% reported seeking acute care for asthma in the past year.^154^ A recent analysis of 12,000 adult respondents to the 2015 Brazil National Health and Wellness Survey showed that 4.1% of respondents had an asthma diagnosis, and among asthmatics, 51.2% had uncontrolled disease, 36.4% had partially controlled symptoms, and only 12.3% were fully controlled.^155^ Adherence to prescribed asthma medication in the public health system in Brazil has been estimated to range from 49.8% among children in primary health care in Belo Horizonte^156^ to 83.9% in adults with severe asthma in Salvador.^157^ Adherence was associated with favourable outcomes in both studies. The definition of severe asthma has changed over time. The current definition adopted by the Global Initiative for Asthma (GINA)^158^ requires laborious and lengthy clinical workup and follow up. There is no reliable population‐based estimate of prevalence of severe asthma in LA using this latest definition^158^ However, an analysis of large European and Brazilian severe asthma cohorts showed them to be remarkably similar in terms of clinical characteristics, despite marked ethnic, socioeconomic, and environmental differences.^159^ Such an observation supports the concept of a single disease with several phenotypes rather a syndrome of multiple diseases.

### 6.3 Asthma costs and implementation of good practice

Direct and indirect economic costs of asthma affect families and health systems but can be reduced by cost‐effective interventions.^160^ In Brazil, a greater proportion of family income is taken up by asthma costs in families with a asthmatic child or adolescent compared to those with an adult with asthma,^161^ while a study in Colombia showed an association between disease severity and family costs that reached up to $2235 annually.^162^ Major efforts have been made to disseminate evidence‐based strategies for asthma management, generally based on the reports of the GINA,^158^ or local adaptations of this report in LA countries.^151^, ^163^ However, major barriers to better asthma management remain, including access to trained physicians and inhaled corticosteroids (ICS), particularly in primary health care of underserved urban populations. To improve asthma care, reference centres have been set up in Brazil and other LA countries. An example is the Programme for Control of Asthma in Bahia (ProAR) in Brazil which set up reference centres for the multidisciplinary care of patients with severe asthma with the provision of free access to medication including combination inhaled ICS with a long‐acting beta_2_ agonist. There was a 74% reduction in hospital admissions due to asthma in Salvador, a city of nearly 3 million inhabitants, following implementation of ProAR.^164^ Asthma can have potentially ruinous costs on family finances,^142^, ^165^, ^166^ and ProAR was shown to have a major impact in reducing direct and indirect economic costs to patients and their families.^143^, ^165^, ^166^

### 6.4 Building capacity of primary health care teams—The way forward

There is a major unmet need in primary health care to improve capacity to manage asthma adequately in LA. ProAR has recently focussed efforts on building the capacity of primary health care workers to manage asthma throughout Brazil, in collaboration with other groups.^167^, ^168^, ^169^ Important barriers for asthma control in LA include the neglect of asthma as a public health issue by health authorities. Despite the provision of free asthma medications in many settings, there is no training of primary health care teams to diagnose and treat asthma. Combined ICS‐LABA inhalers, required for most patients with moderate to severe asthma, generally require a specialist prescription, thus reducing access to best treatment for a large proportion of patients.

## 7 PERSPECTIVES FOR THE FUTURE

Our understanding of asthma in LA has improved substantially over the past 10 years through the efforts of LA researchers alone or through international networks such as SCAALA. An important component of the SCAALA programme has been research training and capacity development fostering the acquisition of expertise in asthma research across traditionally siloed disciplines. During the coming decades, improvements in research capacity and access to research funds within LA countries are likely to see major advances in our understanding of asthma and its causes and prevention.

A key area of asthma research that has emerged over the last 10 years is the understanding that asthma disease includes several phenotypes each of which may be linked to one of more distinct endotypes that themselves have distinct underlying causal mechanisms. Most asthma in a population setting in LA is non‐atopic and is likely to represent a variety of distinct endotypes. For example, non‐atopic asthma may occur through pathways mediated by stress‐linked and neural mechanisms in a context of poverty, inequalities, violence, and inadequate health care. More research is needed to understand these causal pathways, how distinct endotypes may be identified in populations of asthmatics, and how they may differ from perspectives of disease trajectories, prognosis, response to treatment, and, more importantly, potential preventive strategies.

In line with our understanding of the increasing complexity of asthma disease, recent thinking on asthma management has been to stratify patients by the presence of traits (e.g. altered cough reflex, airflow limitation and airways inflammation, and infection) that can be identified and treated.^170^ While such thinking might be more easily applied in HICs, it should be made simple to be feasible in LA, as most asthma is present in poor urban populations with limited access to health care resources. In such settings, particularly in primary health care that must manage most patients, management options when present are constrained by limited time, and scarce resources for diagnosis and medications. Managing this massive disconnect between need and provision is the greatest challenge to asthma care in LA and needs to be prioritized if the burden of asthma is to be reduced in such resource‐poor locations.

## 8 CONCLUSION

LA is now the most urbanized region in the world, where most of the population is packed into unplanned urban spaces in conditions of extreme poverty and lack of access to basic infrastructure and health services. Such an environment is not conducive to good health outcomes and likely contributes to the high prevalence of asthma observed in many urban centres in LA. A variety of evidence from studies done over the past 10 years has helped clarify the role of the environment in modulating the host inflammatory response in marginalized urban settings in LA. These studies have shown that common childhood infections and poor hygiene exposures enhance immune‐regulatory responses and reduce atopy but have unclear effects on asthma symptoms. Our understanding has improved also of how various social and lifestyle factors such as obesity/diet and psychosocial stress/mental illness are associated with asthma. Further, African ancestry, present in many LA populations, enhances asthma risk while a wide variety of genetic factors, many of which have been identified in other regions, can increase risk also. The historical neglect of the African diaspora in the Americas must also be considered, as do associations of African ancestry with asthma as being indicative of genetic rather than social and environmental factors. Associations of asthma symptoms with multiple environmental and genetic risk factors underline the importance of defining disease endotypes and their respective causal mechanisms. Although much of the burden of asthma can be prevented by greater access to adequate disease management including ICS, and reduced exposure to environmental triggers such as tobacco smoke, future interventions for disease prevention will likely require a much better understanding of how specific environmental exposures interact with genetic susceptibility to cause disease.

## AUTHOR CONTRIBUTIONS

Conceptualization: Philip J Cooper, Camila A Figueiredo, Alvaro A Cruz, Mauricio L Barreto. Drafting: Philip J Cooper, Camila A Figueiredo, Alvaro A Cruz, Leticia Marques dos Santos, Rita C Ribeiro‐Silva, Gustavo Costa, Alejandro Rodriguez, Neuza Alcantara‐Neves, Laura C Rodrigues. Literature search: Valdirene Leao Carneiro, Thiago Magalhães, Talita dos Santos de Jesus, Raimon Rios, Hugo Bernardino F da Silva, Ryan Costa, Martha E Chico, Maritza Vaca. Editing and review of final document: all authors.

## CONFLICT OF INTEREST STATEMENT

The authors declare that they have no conflict of interest.

## Data Availability

Data sharing not applicable to this article as no datasets were generated or analysed during the current study.
